# Quantitative Characterization of CD8+ T Cell Clustering and Spatial Heterogeneity in Solid Tumors

**DOI:** 10.3389/fonc.2018.00649

**Published:** 2019-01-07

**Authors:** Chang Gong, Robert A. Anders, Qingfeng Zhu, Janis M. Taube, Benjamin Green, Wenting Cheng, Imke H. Bartelink, Paolo Vicini, Bing Wang, Aleksander S. Popel

**Affiliations:** ^1^Department of Biomedical Engineering, School of Medicine, Johns Hopkins University, Baltimore, MD, United States; ^2^Department of Pathology, Johns Hopkins University School of Medicine, Baltimore, MD, United States; ^3^Bloomberg-Kimmel Institute of Cancer Immunotherapy, Johns Hopkins University School of Medicine, Baltimore, MD, United States; ^4^Department of Dermatopathology, Johns Hopkins University School of Medicine, Baltimore, MD, United States; ^5^Department of Biostatistics, University of Michigan, Ann Arbor, MI, United States; ^6^Clinical Pharmacology, Pharmacometrics and DMPK, MedImmune, Mountain View, CA, United States; ^7^Clinical Pharmacology, Pharmacometrics and DMPK, MedImmune, Cambridge, United Kingdom; ^8^Department of Oncology and Sidney Kimmel Comprehensive Cancer Center, Johns Hopkins University, Baltimore, MD, United States

**Keywords:** digital pathology, spatial patterns, spatial statistics, immuno-architecture, systems biology

## Abstract

Quantitative characterization of the tumor microenvironment, including its immuno-architecture, is important for developing quantitative diagnostic and predictive biomarkers, matching patients to the most appropriate treatments for precision medicine, and for providing quantitative data for building systems biology computational models able to predict tumor dynamics in the context of immune checkpoint blockade therapies. The intra- and inter-tumoral spatial heterogeneities are potentially key to the understanding of the dose-response relationships, but they also bring challenges to properly parameterizing and validating such models. In this study, we developed a workflow to detect CD8+ T cells from whole slide imaging data, and quantify the spatial heterogeneity using multiple metrics by applying spatial point pattern analysis and morphometric analysis. The results indicate a higher intra-tumoral heterogeneity compared with the heterogeneity across patients. By comparing the baseline metrics with PD-1 blockade treatment outcome, our results indicate that the number of high-density T cell clusters of both circular and elongated shapes are higher in patients who responded to the treatment. This methodology can be applied to quantitatively characterize the tumor microenvironment, including immuno-architecture, and its heterogeneity for different cancer types.

## Introduction

The tumor microenvironment (TME) governs tumor development as a result of the interaction between cancer cells, various stromal cells, and immune cells mounting the anti-tumor immune response (1). Thus, deciphering the characteristics of TME, especially its spatial intra-tumoral heterogeneity (ITH), can potentially lead to predictive biomarkers for cancer treatments (2–4). Among characterizations of ITH, immuno-architecture is of particular interest in the setting of mono- or combination cancer therapies involving immune checkpoint blockade, as they reflect the dynamic balance between cancer and immune cells and can provide guidance for interventions to tip this balance (5). The density of tumor infiltrating lymphocytes (TIL) and level of PD-L1 expression, along with their geographical relation to the invasive fronts, are being actively studied as potential biomarker candidates for different cancer types, including melanoma and breast cancer (6, 7).

Digital Pathology is a growing field and has been used in a few specific areas, such as in cancer diagnosis and biomarker analysis for decision making in cancer treatment (8, 9). The process starts with digitization of patient pathology samples, followed by pathological interpretation on a computer screen, or image analysis using algorithms to extract information of interest (10, 11). The benefits of digital pathology are multifold. Using this technique, both data acquisition and interpretation can be more easily standardized, which increase the reproducibility of the analysis. Whole scanned slides give more complete, wider fields of view, views that are not possible with a light microscope. Combined with image registration tools, multiple layers of whole slide images obtained from the same sample can be mapped together, allowing functional annotation with multiple channels and spatial analysis among different labels (12, 13). The available image analysis algorithms have also progressed significantly in recent years. Deep learning and other Artificial Intelligence (AI) algorithms can be trained to perform tasks such as nuclei/cell/gland segmentation and tissue classification, based on which further assessment of disease progression can be made (14, 15). Assisted by modern image analysis tools, pathologists are able to examine patient histological data with higher throughput, and in a more quantitative and objective manner (16). With the increased accessibility of data storage and data transfer bandwidth, the management and sharing of such image data are made much easier, fostering collaborations among researchers from a wider range of fields and locations (17).

However, against the background of the growth in characterization of tumor microenvironment in patient tissue samples with digital pathology and its potential to enhance the area of cancer prognosis and treatment outcome (18–20), the link between disease mechanisms, treatment response and pathological images is still underexplored. An ideal tool to bridge our knowledge of basic cancer biology and cancer histopathology clinical data is computational systems biology, which can mechanistically integrate multiple components of the system to reproduce spatio-temporal dynamics of tumor development (21). In particular, agent-based models (ABM) incorporate local interactions between cells and the environment in a computer simulation to generate and analyze the global emergent properties of the tumor microenvironment and its spatial heterogeneity (22–26). Due to the explicit spatial nature of such methods, comparisons between pathological images and model outputs can be readily achieved, providing a means to setup initial conditions for the computational models, as well as calibrate and validate them. Two different approaches are available to achieve this goal: first, after image segmentation, the detected objects such as cancer cells and immune cells can create an exact mapping for the corresponding agents in model simulation (27); second, further quantification of the spatial patterns can be performed based on the segmentation output, which is then used for parameter estimation or compared with the same quantification of model-generated images (28–30). Because the second approach makes comparisons of parameters, it takes into account the potential uncertainty arising from limited tissue sampling, and can thus benefit from reduced risks of overfitting and an increased predictive power. Such quantification is applicable at multiple biological scales; immuno-architecture mainly corresponds to tissue-scale patterns formed by collections of individual cells.

In order to characterize the spatial heterogeneitry of the tumor microenvironment with respect to clusters of cells, various metrics can be used. For example, morphometric analysis can help quantify the shape of cell clusters (31); using spatial point pattern analysis, one can quantify the underlying interaction between cells leading to such patterns (32, 33).

In this study, we developed a computer-aided workflow to quantitatively characterize the spatial heterogeneity of tumor microenvironment, and apply this framework to digitalized histopathology data generated using whole slide imaging of tissues from patients in a clinical trial on PD-1 blockade in cancer with mismatch repair deficiency (34). In that clinical trial, intratumoral tissue-infiltrating lymphocytes (TIL) CD8+ T cell density is found to associate with a trend toward objective response, which motivates us to further look into the spatial heterogeneity of T cell distribution and search for measurements with higher predictive power for patient responsiveness to PD-1 blockade therapy. In this workflow, image analysis algorithms are employed to identify and locate CD8+ cells in the patient tissue sample, mapping out spatial point patterns for cells of interest. The spatial point patterns are then subject to spatial point process model fitting and morphometric analysis to extract information regarding interactions among cells as well as the geometric properties of cell clusters arising from such interactions. Using this workflow, digitized image data are converted to quantitative and interpretable measurements, which are essentially a high-level representation of intra- and inter-tumoral heterogeneity. These metrics can then help guide the construction, calibration and validation of systems biology models for immuno-oncology research and clinical applications. We also explore how the metrics generated from the analysis could suggest predictive biomarkers of responsiveness to immune chekcpoint blockade treatment for patients.

## Methods

**Figure 1:**
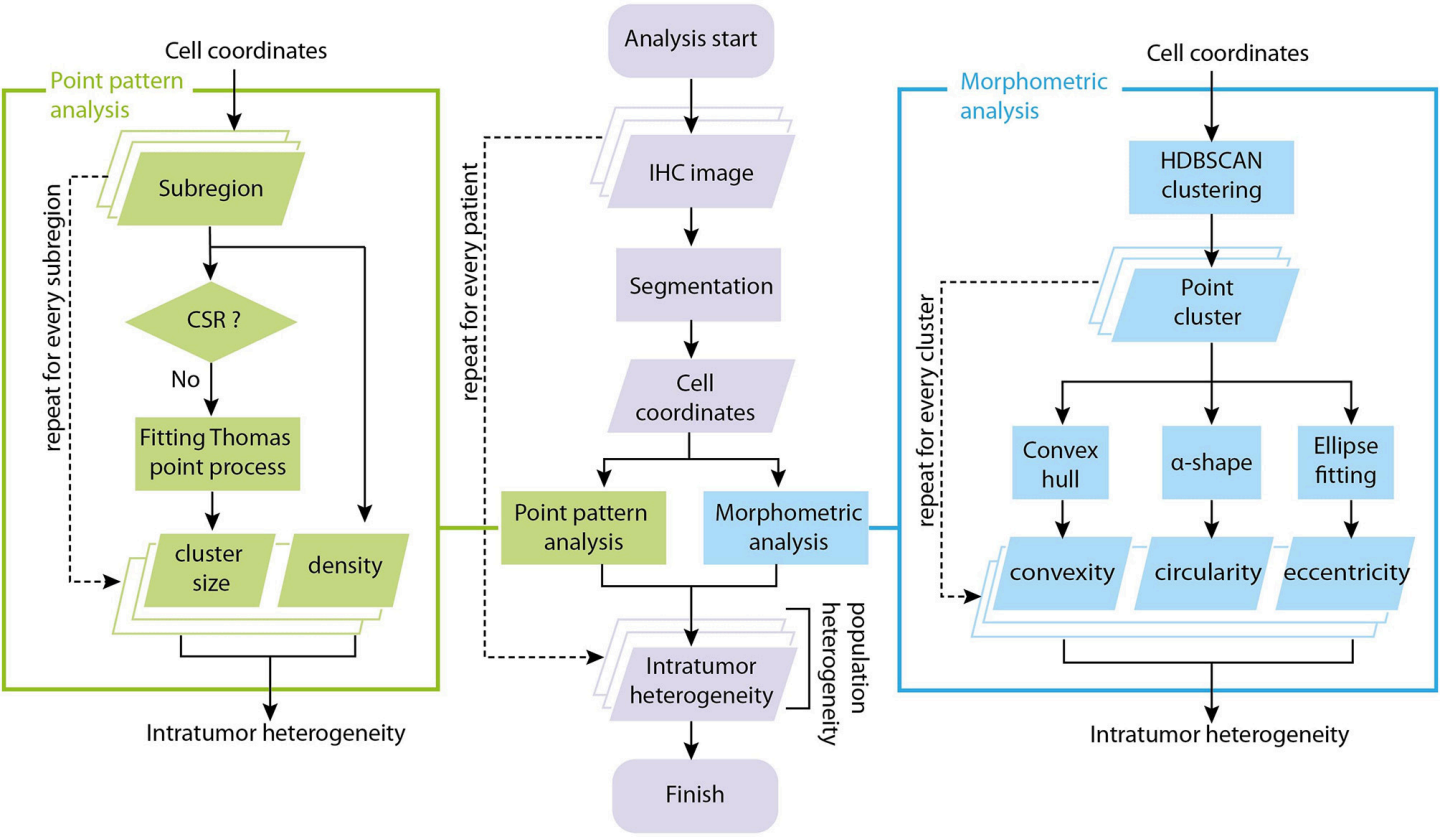
Overall workflow of tumor spatial heterogeneity quantification. The analysis starts with brightfield IHC with CD8+ staining. For each patient slide, we perform image segmentation to obtain coordinates of CD8+ T cells. The coordinate list from each slide is fed to two sub-procedures: spatial point pattern analysis and morphometric analysis. In spatial point pattern analysis, local patterns are obtained using a moving window, tested for complete spatial randomness (CSR) and fitted to a clustering point process model if it is aggregated. In morphometric analysis, the full coordinate map is divided into clusters, and a series of shape descriptors are calculated for each cluster. The arrays of shape descriptors and fitted parameters constitutes a quantitative representation of the intra-tumoral (intra-slide) heterogeneity for that patient. We repeat the process on each slide to obtain measures of the inter-tumoral (inter-slide) heterogeneity.

The input data to our analysis are brightfield immunohistochemistry (IHC) images with CD8+ staining of tissue excised from patients with mismatch repair deficient colorectal cancer, mismatch proficient colorectal cancer or mismatch deficient noncolorectal cancer (34) prior to the onset of PD-1 based Immunotherapy (pembrolizumab). For each patient slide (*N* = 29 patients with known outcomes and successful CD8+ staining, one slide is available for each patient), we performed image segmentation to obtain coordinates of CD8+ T cells. The locations of T cells from each slide are analyzed using two methods: spatial point pattern analysis and morphometric analysis. In spatial point analysis, the full point pattern is divided into sub-regions, and each sub-region is tested for complete spatial randomness (CSR) and fitted to a point process model if CSR is rejected. In morphometric analysis, cluster analysis is performed for the full point pattern map, and a series of shape descriptors are calculated for each cluster. The arrays of fitted parameters from spatial point analysis and shape descriptors from cluster analysis constitute a quantitative representation of the intra-tumor heterogeneity. We then repeat the process for each slide to obtain information on population variation and calculate inter-tumor, or inter-patient heterogeneity. The overall workflow is shown in Figure 1.

### Segmentation of Patient IHC Pathological Slides

The original images are in Aperio format (.svs), where CD8+ T cells are stained using immunohistochemistry. The images received for the computational analysis have been fully de-identified. The methods for staining the tissue are described in Le et al. (34). Briefly, the expression of CD8 diaminobenzidine (DAB)-stained cells was evaluated within the tumor and at the invasive fronts of the tumor in an immunohistochemical analysis. The CD8-stained slides were scanned at 20x equivalent magnification (0.49 micrometers per pixel) on an Aperio ScanScope AT.

We use the software HALO (v2.2.1870.31) from Indica Labs (Corrales, NM) to perform segmentation of digitized pathological images, using the module “Indica Labs–CytoNuclear v1.6.”To evaluate the performance of our segmentation algorithm, manual segmentation is performed in randomly selected subregions of each patient slide. Samples (*N* = 100) are taken from each slide using systematic sampling scheme and CD8+ T cells are manually identified and compared with CD8+ T cells detected with HALO. The number of cells detected both manually and by software (true positive, TP) is denoted with *x*_*i, j*_, where *i* indicates the patient and *j* indicates the sample. The number of cells identified manually (including both TP and false negative, FN, which are missed by algorithm) or by software (including both TP and false positive, FP, which are detected by algorithm but rejected in manual approach) are denoted with *m*_*i, j*_ and *n*_*i, j*_, respectively. The overall Sensitivity (recall rate, R) and Precision (P, or 1-false discovery rate) of the segmentation algorithm and their standard error of mean are expressed as:

(1) $$R=\frac{TP}{TP+FN}=\frac{\sum_{i,j}x_{i,j}}{\sum_{i,j}m_{i,j}}$$

(2) $$P=\frac{TP}{TP+FP}=\frac{\sum_{i,j}x_{i,j}}{\sum_{i,j}n_{i,j}}$$

(3) $$se_{R}=\sqrt{\frac{R(1-R)}{TP+FN}}$$

(4) $$se_{P}=\sqrt{\frac{P(1-P)}{TP+FP}}$$

The numerical estimates of the segmentation process are presented in the Results section below.

### Spatial Point Process Model Fitting

Spatial statistical analysis is performed on point pattern of CD8+ T cells, which is created from coordinates obtained from image segmentation. We define local point pattern as the pattern of the subset of CD8+ T cells within a sub-regions of the full image. Sub-regions are taken using a rectangular moving window with edge lengths of *x*_*window*_ and *y*_*window*_ and step size of *x*_*step*_ and *y*_*step*_. The window size should be large enough so that each window properly include local density variability required for analysis, while at the same time not too big so that we can make the assumption that the underlying spatial point process is stationary (Figure S1). In this study, we set the window and step size to be *x*_*window*_ = *y*_*window*_ = 0.5 *mm* and *x*_*step*_ = *y*_*step*_ = 0.25 *mm*.

Each local point pattern is first tested for Complete Spatial Randomness (CSR) using Clark-Evans test (one-tailed, *H*_*A*_: clustered distribution, siginificance level α = 0.05) (35). If the pattern diverges significantly from a homogeneous Poisson point process and is thus deemed to show aggregation, we fit a Thomas point process model to the local point pattern and record the fitted parameter values (36–38). In doing this, we are assuming a two-stage process to generate the point pattern: in the first stage, a homogeneous Poisson process with intensity κ determines the parent points within the sub-region; and in the second stage, child points are generated with another Poisson process of intensity μ and location determined following an isotropic Gaussian distribution centered at each parent point with variance σ^2^. The theoretical Ripley's K function describing the expected number of other points within distance *r* of any chosen point divided by intensity is:

(5) $$K(r)=\pi r^{2}+\frac{1}{\kappa }(1-e^{-\frac{r^{2}}{4\sigma^{2}}})$$

For each sub-region, we obtain the fitted parameters κ, μ, and σ, and use them to interpret the clustering of CD8+ T cells within this sub-region. κ is the number of CD8+ T cells clusters per unit area; μ is the number of CD8+ T cells within each cluster. Because the second stage is an uncorrelated bivariate Gaussian with 0 mean and equal variance, the distance of each cell toward the cluster center, *r*, follows a Rayleigh distribution $\frac{r}{\sigma^{2}}e^{-\frac{r^{2}}{2\sigma^{2}}}$, whose first moment (average distance) can be calculated as $\mu \left(r\right)=\sigma \sqrt{\pi /2}$, and the radius of the circle covering 95% points of each cluster can be calculated as $r_{0.95}=\sigma \sqrt{2ln\left(20\right)}$.

The CSR testing and model fitting are performed using function “kppm” from R package “spatstat,” with cluster model argument “Thomas” (39).

### Cluster and Morphometric Analysis

Cluster analysis is performed to directly identify CD8+ T cell clusters from the point pattern generated from image segmentation. We use the clustering algorithm Hierarchical DBSCAN (HDBSCAN) (40) to extract CD8+ T cell clusters. This method constructs cluster hierarchy from density-adjusted distance connectivity, and extracts clusters by comparing parent and child cluster stabilities. R package “largeVis” (41) is used for cluster analysis, in which a variation of the HDBSCAN algorithm is implemented. Parameters used for HDBSCAN are minPts = 30 (minimum point per cluster) and *K* = 4 (core neighborhood range).

Each CD8+ T cell cluster identified in the aforementioned cluster analysis is subject to morphological analysis to obtain quantification of the cluster shapes. We determine the shape of a cluster with alpha-shape, in which the edges are defined as the set of segments between each pair of points that are located on circles of a given radius α. Alpha-shapes are calculated using R package “alphahull” (42). For each cluster, we increase the α value starting from 10 μ*m* until the alpha-shape is one single connected region containing all the points from the cluster. After obtaining the alpha-shape, we calculate various metrics for each cluster, as follows.

Convexity:

(6) $$f_{conv}=\frac{A_{\alpha }}{A_{conv}},$$

where *A*_α_ is the area of the alpha-shape and *A*_*conv*_ is the area of the convex hull. Circularity:

(7) $$f_{circ}=\frac{4\pi A_{\alpha }}{P_{\alpha }^{2}},$$

where *P*_α_ is the perimeter of the alpha-shape.

We also fit ellipses to each cluster. Assuming the points within one cluster follow bivariate normal distribution X~N(μ,Σ), the ellipse can be constructed as a contour of a given confidence level (1-α), e.g., 95% CI, whose semi-major and -minor axis lengths can be calculated as:

(8) $$a=\sqrt{\lambda_{1}\chi_{2}^{2}(\alpha )}$$

(9) $$b=\sqrt{\lambda_{2}\chi_{2}^{2}(\alpha )},$$

where λ_1_ and λ_2_ are eigenvalues of Σ, and λ_1_ ≥ λ_2_. Thus, eccentricity can be calculated as:

(10) $$e=\sqrt{1-\frac{b^{2}}{a^{2}}}=\sqrt{1-\frac{\lambda_{2}}{\lambda_{1}}}$$

### Measuring Intra- and Inter-tumoral Heterogeneity

The aforementioned metrics from both spatial point process model fitting and morphometic analysis can be utilized to gauge the spatial heterogeneity within a patient slide (intra-tumoral) as well as among different patients (inter-tumoral). In the point pattern analysis step, the statistics obtained from each window reflect local spatial properties of CD8+ T cell at the location of that window. In the morphometric analysis, the shape and density of CD8+ T cell clusters in different regions of the slide is assessed.

Without assuming a distribution of these assessments, we use quartile coefficient of dispersion (QCoD) to evaulate their variability across a slide to calculate the intra-tumoral heterogeneity. Similarly, for each patient slide, we use the median value of each metric as a proxy for that patients's overall CD8+ T cell spatial property, and calculate the QCoD among all the patients as the inter-tumoral heterogeneity. QCoD is calculated using the following formula:

(11) $$QCoD=\frac{Q_{3}-Q_{1}}{Q_{3}+Q_{1}},$$

where Q1 and Q3 are the first and third quartiles of each metric.

### Correlating Morphological Metrics to Treatment Outcomes

Each tumor slide contains regions corresponding to different tissue context. Regions corresponding to tumor and normal tissue were annotated on separate layers using Aperio ImageScope v12.1.0.5029. Computer algorithms are used to determine the invasive front by drawing a band of 250 micron width on both sides of the edge of the tumor where it meets with the non-tumor regions. Because not every patient's tissue image has all three types of regions (tumor, normal, and the invasive front), we define regions of interest (ROI) for each patient sample so that comparison can be made across all patients: the ROI includes all the invasive fronts within a slide if any such region is present; or all the tumor regions if invasive front is not found but tumor regions exist; or all the normal regions if neither invasive front nor tumor region exists. We map this information onto segmentation results to determine intra-tumoral, peritumoral or stromal tumor infiltrating lymphocytes, defined as CD8+ T cells within a tumor region, invasive front, or a non-tumor region, respectively. For each cluster, if any of its cell is peritumoral, the cluster is considered as invasive front associated; otherwise, if the cluster has intra-tumoral CD8+ T cells, this cluster is considered tumor associated; the rest of the clusters are normal tissue associated.

For each patient, the responsiveness to the immunotherapy during the clinical trial is available in the format of Response Evaluation Criteria in Solid Tumors (RECIST). The trial includes patients who are either mismatch-repair deficient and microsatellite instable (MSI), or mismatch-repair proficient and microsatellite stable (MSS) (34). For the MSI cases, the outcomes of complete response and partial response are considered responsive to treatment; patients with a stable or progressive disease are considered non-responders because further mutations are likely to arise to trigger a relapse. On the contrary, for MSS patients, stable disease can be considered responsive to the treatment. We group the patients into responders and non-responders regardless of their intial cohort designations, and compare how different metrics separate patients between the two groups. We use nonparametric Wilcoxon rank-sum test for this task, due to the small number of samples and the lack of evidence to assume normal distribution.

## Results

### Image Segmentation

**Figure 2:**
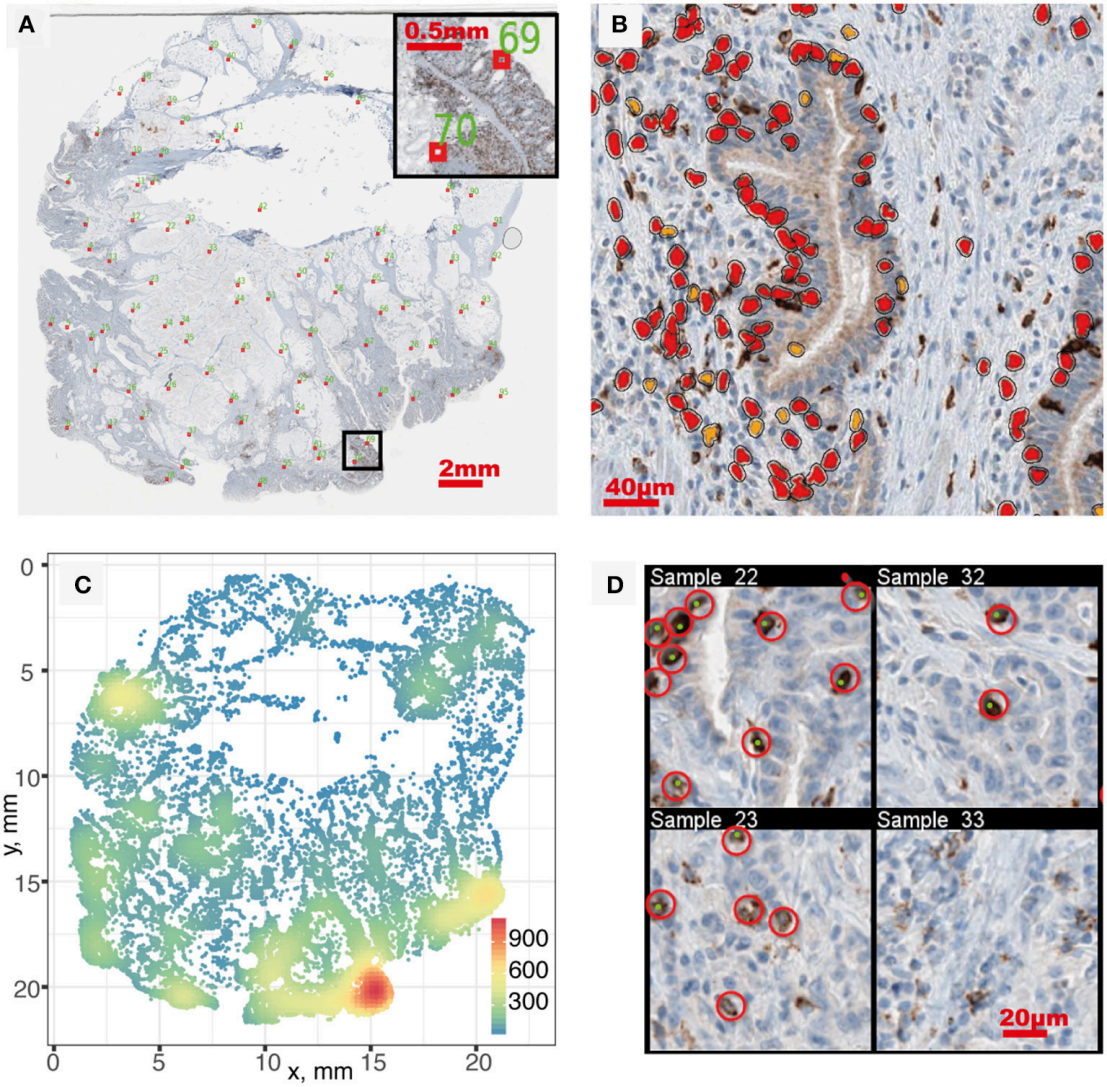
Image segmentation and segmentation quality evaluation. **(A)** IHC whole slide, red boxes indicate sampled location for quality evaluation. **(B)** Segmentation result of a sub-region. Orange and red correspond to positive staining with thresholds of OD > 0.5 and OD > 0.7, respectively. **(C)** Full point pattern. Color indicates cell density (mm^−2^). **(D)** In each sampled region, manual identification of CD8+ T cells (red circles) is performed and compared with segmentation result (green dots) to evaluate performance. Four sampled windows are shown.

We started the analysis with brightfield IHC imaging results from 29 slides from patients stained for CD8 (34). A low-resolution version of one of these images is shown in Figure 2A. Using the software HALO, we performed image segmentation and obtained the coordinates of CD8+ T cells within each IHC image. Figure 2B shows one snippet of the original image superimposed with detected CD8+ T cells. The coordinates of detected CD8+ T cells in the full image are obtained from the segmentation, as shown in Figure 2C. The number of CD8+ T cells identified in each slide ranges from 98 to 106,271, with the median count 11,371. Detailed CD8+T cell counts and densities are listed in Table S1.

After segmentation, we evaluated the performance by sampling the original patient IHC imaging result and comparing the segmentation outcome with manually identified CD8+ T cell location. Twenty samples are taken from each patient slide following the Latin Hypercube Sampling (LHS) scheme (shown as red boxes in Figure 2A) and CD8+ T cells are manually identified, as shown in Figure 2D. From the 620 sample regions, we detected the following number of cells for different categories: *TP* = 2,904; *FN* = 1,010; *FP* = 394. Using Equations 1–4, we can calculate the point estimate and their standard error to be Recall *R*(%) = 74.2±0.7 and Precision *P*(%) = 88.1±0.6. To assess the level of agreement between the segmentation outcome by HALO and that by manual counts, the Spearman's rank correlation coefficient was calculated between the number of CD8+ cells by HALO (i.e., TP + FP) and the number of CD8+ cells by manual identification (i.e., TP + FN). The estimate is 0.985, which indicates a very strong relation between these two outcomes. Most of the false negatives happen in regions with very high density of CD8+ T cells due to the limitation of the segmentation algorithm; however, the detected density in these regions is still much higher compared to other regions despite the underestimation.

### Tumor Spatial Heterogeneity Assessed With Spatial Point Process Models

**Figure 3:**
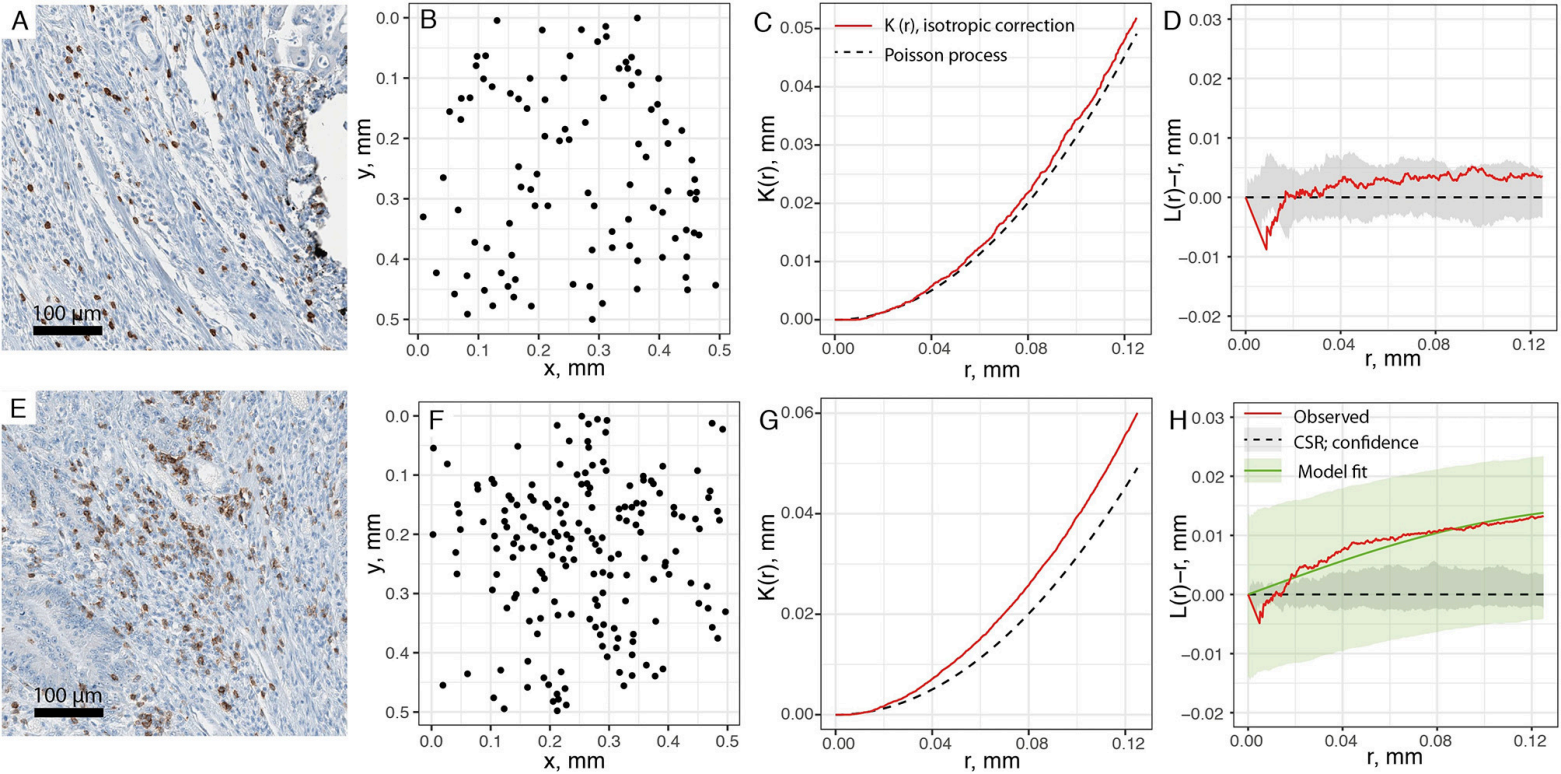
Estimating spatial statistics from sub-regions of point pattern. A moving window is used to quantify sub-regions of each slide, with edge length of 0.5 mm and steps of 0.25 mm. **(A)** Original IHC sub-region. **(B)** Point pattern within the same sub-region. **(C)** Ripley's K estimation using isotropic edge correction for pattern in **(B)**. **(D)** L-transformation of K function (red). When testing for CSR, no significant aggregation pattern is detected, thus no clustering model fitting is performed for pattern **(B)**. Black dashed line is theoretical value of L(r)-r for Poisson process (random), and gray envelope is 95% confidence interval obtained from Monte-Carlo simulations. **(E–H)** is the same process performed over another region. Pattern F is tested as aggregated, and fitted to modified Thomas process model (green line). Green envelope is the global envelope from Dao-Genton goodness-of-fit test.

With the spatial point patterns of CD8+ T cells obtained from segmentation and its quality evaluated, we use two different methods to quantify the spatial characteristics of these patterns, as shown in Figure 1. In the first approach, we examine the pattern from each patient with a moving window. For each window, we estimate the first-order property, i.e., the intensity for each window, and test whether the point pattern within the window is random or aggregated (Figures 3A–D). If Complete Spatial Randomness (CSR) is rejected for that window (Figures 3E–H), we further characterize the point pattern by fitting a spatial point process model to it. In this study, we use modified Thomas model for this procedure to reveal the properties of clusters behind the patterns. The fitted parameters for each window serve as quantification for the local characteristics of the point patterns. Some of these parameters are directly used, such as μ from Equation 5, which indicate the number of CD8+ T cells per cluster; others can be transformed for more straightforward interpretation, such as σ^2^, from which we calculate the average distances to cluster center and cluster areas.

As such quantifications are performed in a sliding window across each patient slide, and for every metric taken into consideration we obtain an array of values corresponding to different spatial locations within the patient sample. The variability within each array captures the intra-tumor spatial heterogeneity with regard to that metric. Furthermore, the differences across the probability density distribution of each metric among patients can be used to evaluate the inter-tumoral, population scale heterogeneity.

**Figure 4:**
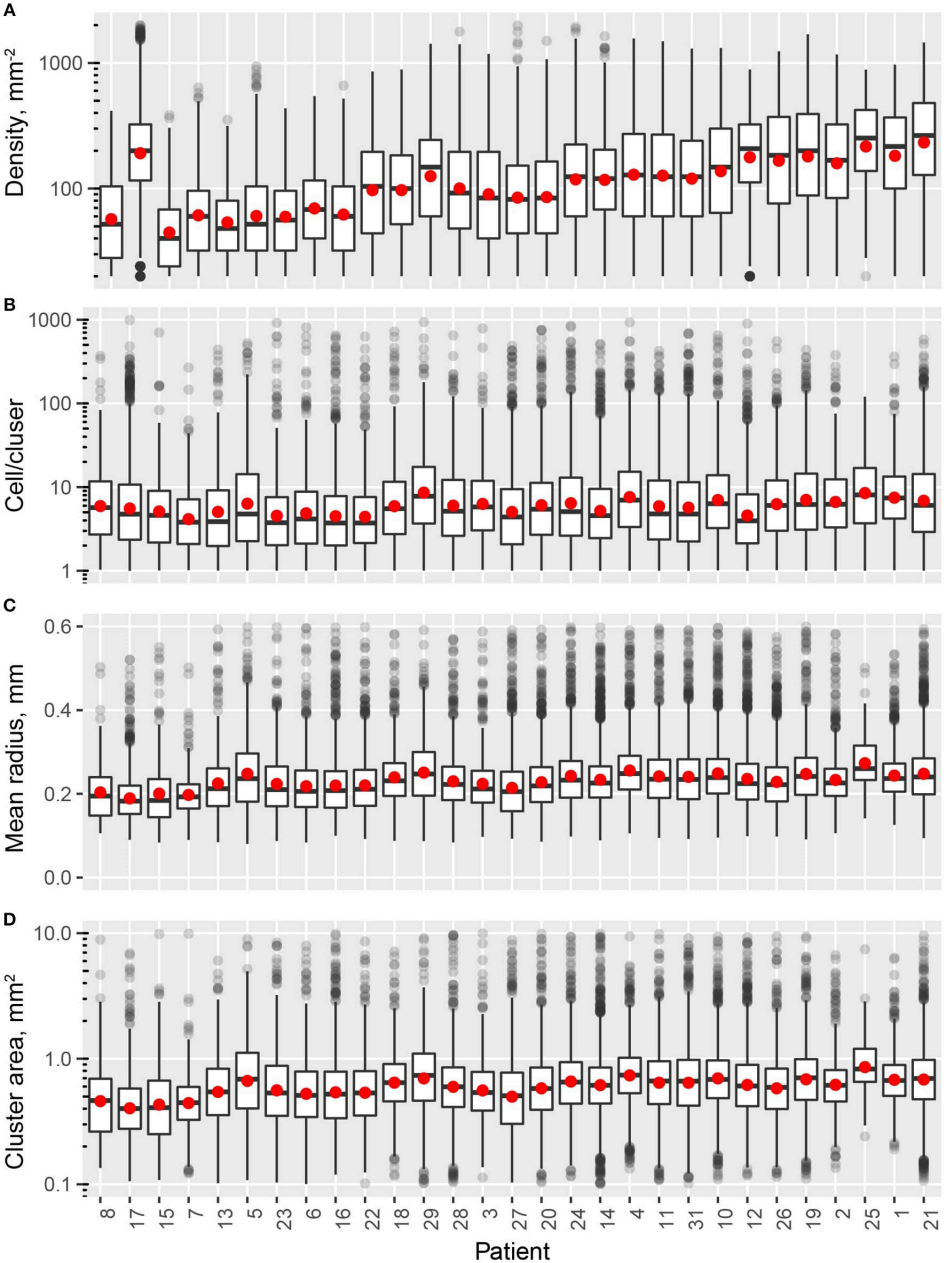
Intra- and inter-tumor heterogeneity quantification using spatial statistics. Patients are sorted by overall CD8+ T cell density. In the box plots, upper and lower hinges indicate 25th and 75th percentiles, respectively. Red dot indicate the mean value. **(A)** Cell density within each window. **(B)** Cell/cluster is fitted parameter from modified Thomas process model. **(C)** Mean distance is the average distance from each point to cluster center. **(D)** Cluster area is calculated at 95% level of the bivariate normal distribution.

In this study, we quantified the aggregation pattern of CD8+ T cells from patients using the following metrics: CD8+ T cell density, number of CD8+ cells per cluster, and the average radius and area of clusters from each window. The results are shown in Figures 4A–D, where the range of each measurement is captured in a Box plot for each patient. The patients are sorted in the order of the overall density of CD8+ T cells in the entire slide. As shown in Figure 4, the number of CD8+ T cells in each cluster range from a few to hundreds, with the medians being <10. The medians of mean distance to cluster center are between 0.2 and 0.3 mm, and the median area of clusters are between 0.5 and 1 mm^2^. For all the four metrics shown here, the variability within each patient sample is high; however, except CD8+ T cell density, the other three examined metrics are relatively consistent across the population in terms of median value and IQR. The quartile coefficient of dispersion (QcoD) within a slide and the QCoD of the median values for each metric across slides are summarized in Table 1.

**Table 1:** Intra- and inter-tumoral QCoD for spatial statistics.

**Metrics**	**QCoD, intra-tumoral (%)**	**QCoD, inter-tumoral (%)**
Density	46.7–68.2	50.0
Cell/cluster	55.1–76.0	22.1
Mean distance	14.1–27.1	5.65
Cluster area	27.6–50.4	11.3

### Tumor Spatial Heterogeneity Assessed With Cell Cluster Morphometrics

**Figure 5:**
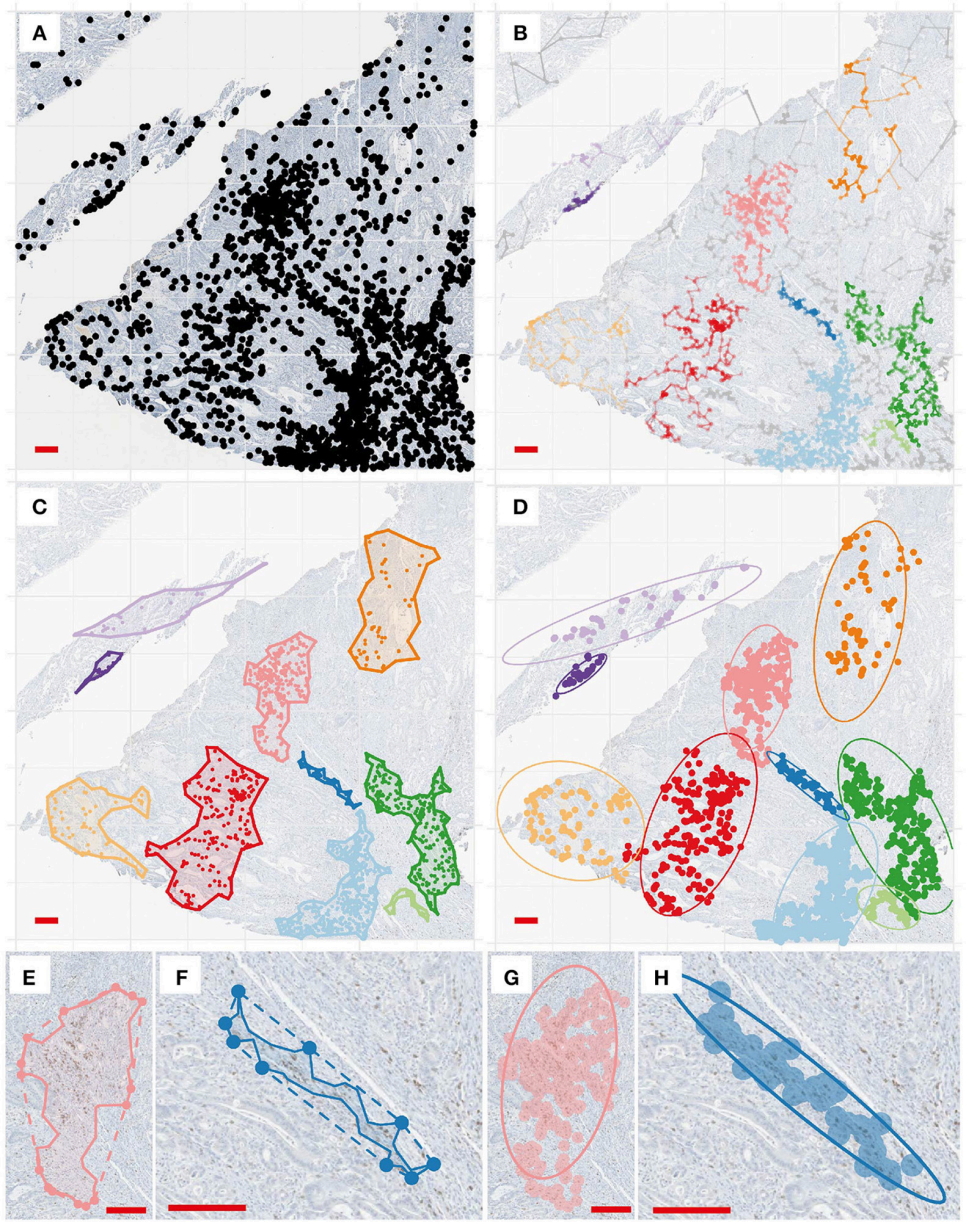
Cluster analysis and shape descriptors. **(A)** Point pattern of a region for illustration. **(B)** Clustering results. Each color correspond to a detected cell cluster. **(C)** For each cluster, we determined the alpha-shape (concave outline). **(D)** For each cluster, ellipse fitting is performed at a 95% level. **(E,F)**. Convex hull (dashed line) and concave shapes of two selected clusters. Convexity is calculated to be 0.76 and 0.41, and circularity is calculated to be 0.42 and 0.12, respectively. **(G,H)**. Ellipse fitting for the same clusters. Eccentricity is calculated to be 0.88 and 0.99.

The second approach we took in this study to evaluate tumor spatial heterogeneity involves two main steps: first, we detected CD8+ clusters from the full CD8+ point patterns, as shown in Figures 5A,B; after that we determine the alpha-shape (Figure 5C) and fitted ellipse (Figure 5D) characteristics and perform morphometric analysis for detected clusters in patients slides. By comparing the concave and convex hulls generated from the subset of points of a cluster, we calculated convexity (Equation 6) and circularity (Equation 7) of a cluster. Using ellipse fitting, we evaluated the elongation of a cluster by calculating its eccentricity (Equation 10). These shape descriptors can be further utilized to categorize the immuno-architecture of the clusters: highly elongated or concave CD8+ T cell clusters (Figures 5F,H) are likely to belong to an invasive front as opposed to a circular one (Figures 5E,G).

**Figure 6:**
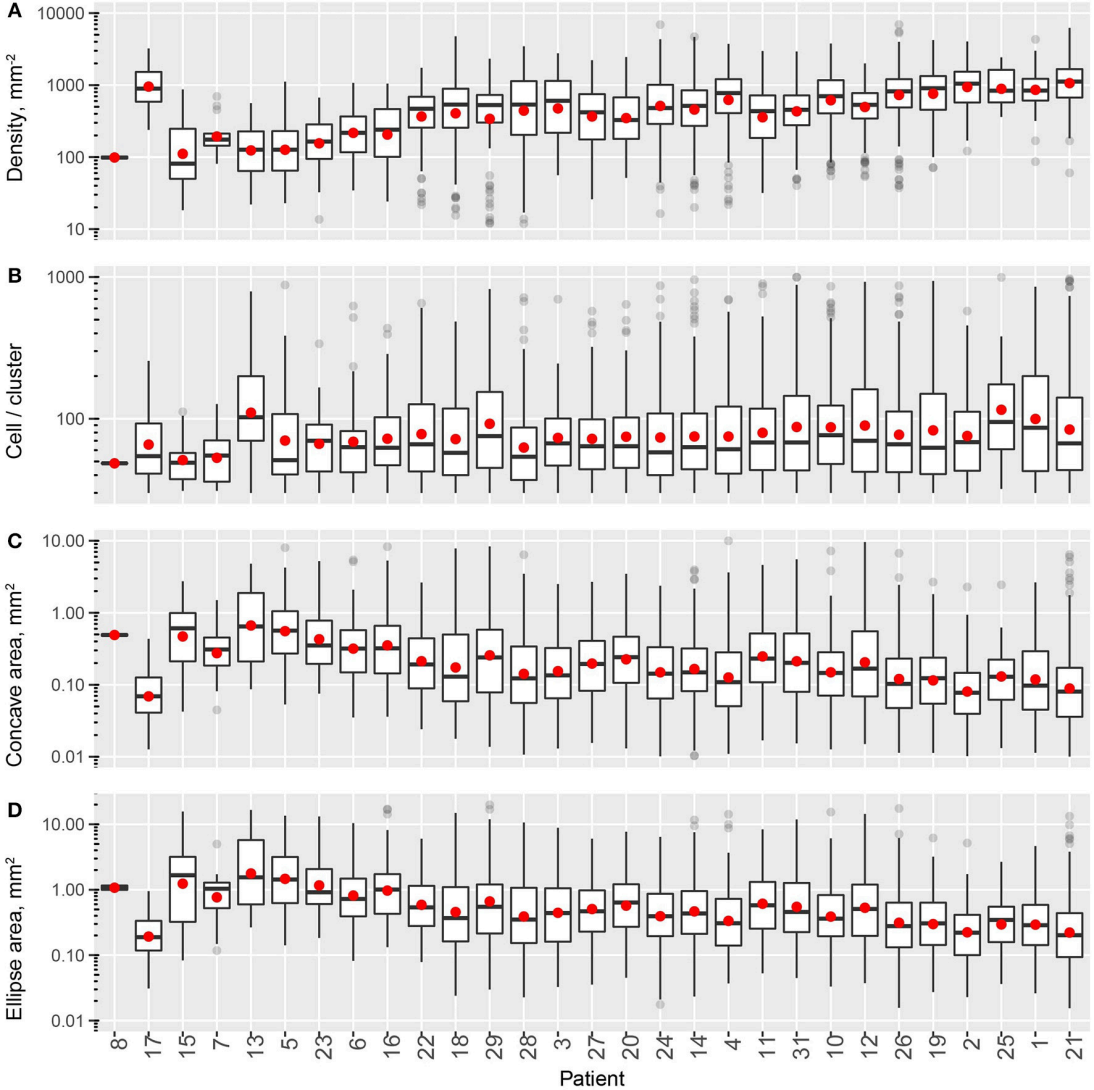
Intra- and inter-tumoral heterogeneity quantification using cell cluster statistics. Patients are sorted by overall CD8+ T cell density. In the box plots, upper and lower hinges indicate 25th and 75th percentiles, respectively. Red dots indicate the mean value. **(A)** Within-cluster density of CD8+ T cells. **(B)** Number of cells per cluster. **(C)** Cluster area, measured as areas of alpha-shape. **(D)** Cluster area, measured as the area of 95% level fitted ellipses.

**Figure 7:**
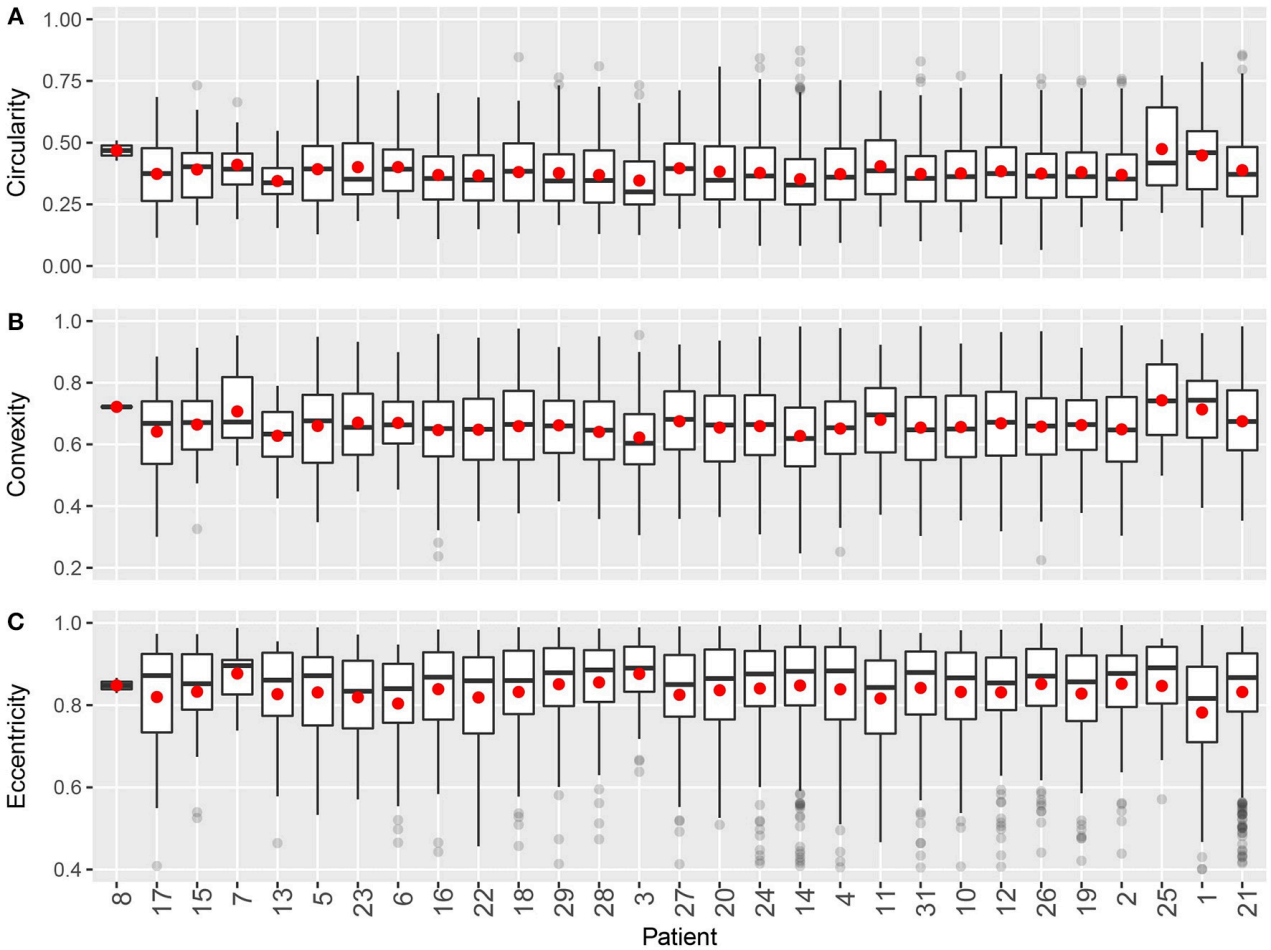
Intra- and inter-tumoral heterogeneity quantification using cluster shape descriptors. Patients are sorted by overall CD8+ T cell density. In the box plots, upper and lower hinges indicate 25th and 75th percentiles, respectively. Red dot indicates the mean value. **(A)** Circularity. **(B)** Convexity. **(C)** Eccentricity.

In this process, multiple measurements are taken for each cluster, including cluster properties (Figure 6) and shape descriptors (Figure 7). The distribution of the values for each metric taken from all the detected clusters in one patient can represent one measurement of intra-tumoral spatial heterogeneity, and inter-tumoral population scale heterogeneity can be evaluated by comparing such distributions across patients. For cluster basic properties, we included the following metrics in our analysis: CD8+ T cell density within clusters, number of cells per cluster, and cluster areas calculated using α-shapes and fitted ellipses. Patients are sorted in the order of increasing overall CD8+ densities. As shown in Figure 6, the median density of cells within clusters ranges 100–1,000 mm^−2^ and is positively correlated with the overall density. The number of cells per cluster is relatively consistent across patients, but is quite heterogeneous within the same slide, ranging from a few tens to hundreds. The median area of each cluster ranges from 0.1 to 0.5 mm^2^ (measured from α-shape) or 0.2–1.2 mm^2^ (measured from fitted ellipses), and correlates negatively with overall density.

For shape descriptors, we measured circularity, convexity and eccentricities of each cluster and plotted their intra- and inter-tumoral heterogeneities. As shown in Figure 7, median circularity ranges from 0.3 to 0.4; median convexity ranges from 0.6 to 0.7; median eccentricity ranges from 0.8 to 0.9. Those metrics are uncorrelated with overall CD8+ T cell density, and are relatively consistent across the population. The quartile coefficient of dispersion (QCoD) within a slide and the QCoD of the median values for each metric across slides are summarized in Table 2.

**Table 2:** Intra- and inter-tumoral QCoD for cluster statistics and shape descriptors.

**Metrics**	**QCoD, intra-tumoral (%)**	**QCoD, inter-tumoral (%)**
Density	2.9–69.6	56.7
Cell/cluster	0.52–65.0	9.4
Alpha-shape area	2.4–86.7	44.0
Fitted ellipse area	7.6–84.2	46.4
Circularity	4.4–32.6	5.3
Convexity	0.19–16.9	1.7
Eccentricity	1.1–11.8	1.2

### Tumor Heterogeneity Quantification Improves Prediction of Immunotherapy Treatment Outcome

**Figure 8:**
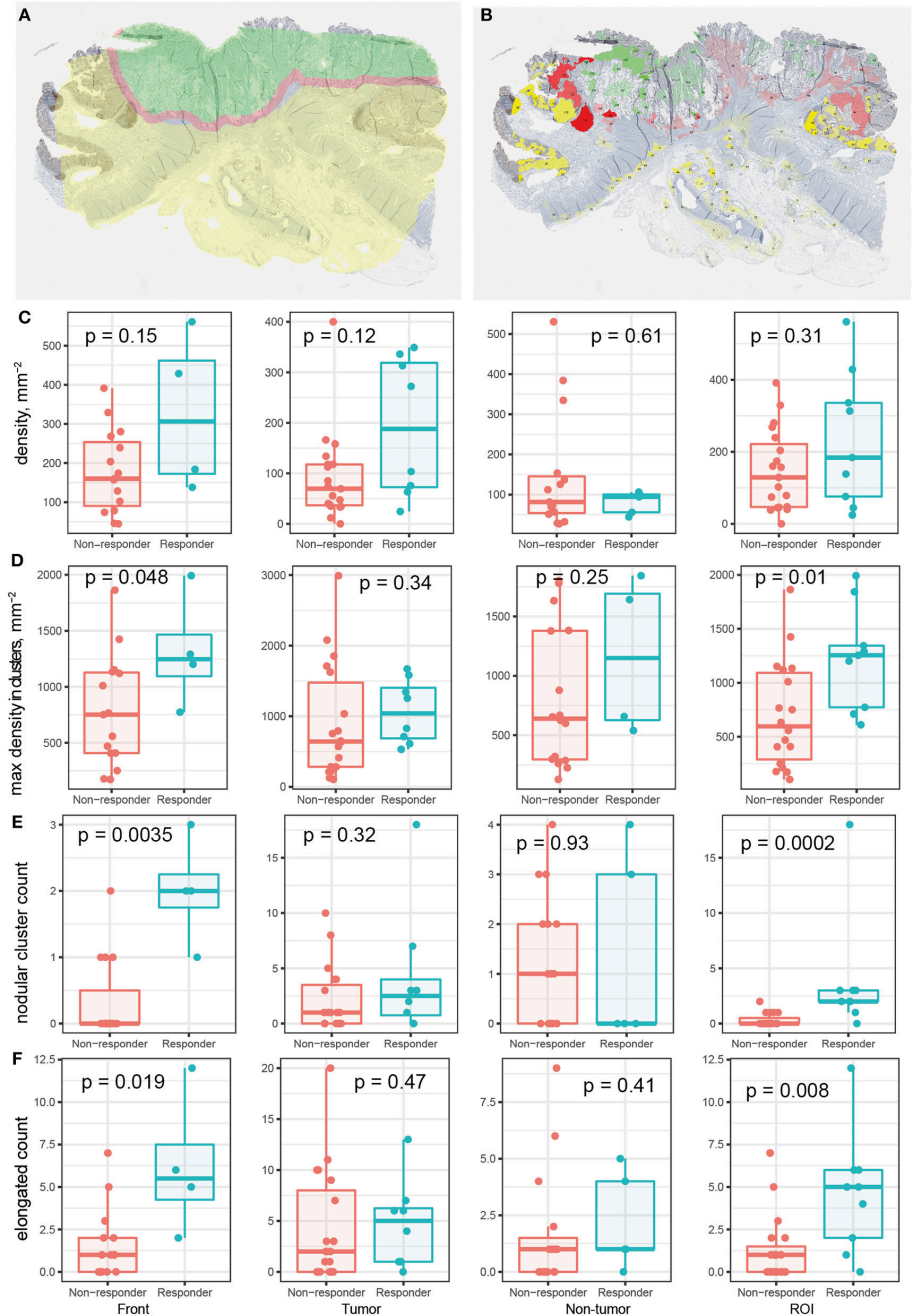
Relationship between region specific metrics and responsiveness to immune checkpoint blockade therapy. **(A)** Patient tissue annotation. Green: tumor; yellow: normal tissue; red: invasive front. **(B)** CD8+ T cell cluster tissue association. Transparency indicates density in each cluster. **(C–F)** Various metrics in different tissue regions for responders vs. non-responders. **(C)** Average CD8+ T cell density. **(D)** Maximum cluster density. **(E)** Number of circular clusters. Criteria: eccentricity < 0.8 AND convexity >0.8 AND circularity > 0.5. **(F)** Number of elongated/irregular clusters. Eccentricity > 0.9 OR convexity < 0.3 OR circularity < 0.3.

With shape descriptors calculated for each cluster, we can use such information to characterize each patient and determine if these descriptors can add to the predictive power of existing biomarkers for treatment outcomes. Metrics of cell markers restricted to certain tissue contexts have been found to better predict treatment outcomes, thus we performed tissue annotation to decide which tissue context is each T cell located: tumor, normal, or the invasive front (Table S1), (Figure 8A). Based on this, we determined the tissue association of the cell clusters using criteia introduced in section Correlating Morphological Metrics to Treatment Outcomes (Figure 8B).

For 28 out of the 29 patients whose CD8+ stainings are analyzed, the treatment outcome from the trial is available (Table S1). We examined the ability of various metrics to differentiate responders from non-responders among the patients. We first checked the density of CD8+ T cells within each region. The results are shown in Figure 8C; CD8+ T cell densities in none of the regions are different for responders and non-responders. Then we take into account information we obtained in the previous section. We first focus on the maximum of densities within clusters (excluding very small clusters, e.g., clusters with less than 50 cells) for each patient. The density within invasive front and ROI are significantly higher in responders compared to non-responders (α = 0.05) (Figure 8D); the results for tumor and non-tumor tissue are not significant. These results indicate that using this measurement, if we focus on the invasive front (or tumor/normal regions if no invasive front is identified), the highest density of CD8+ T cell found is likely to correlate with reponsiveness.

We then incorporated the shape descriptors to look for more factors that could be predictive of treatment outcomes. We searched for clusters of the following two types and counted their numbers in different regions of each patient sample: (1) High density, circular clusters that resemble the shape of lymphoid folicles; (2) High density, elongated and irregular clusters. Between MSS and MSI patient groups, those metrics are significantly higher in tumor center, but not in the invasive front (Figure S2). We tested the difference of the number of qualifying clusters in patients who do or do not respond to the treatment, and results are shown in Figures 8E,F. The results indicate that the number of cell clusters of both types are higher in invasive front and ROI for patients who respond better to the treatment. As a comparison, the total number of CD8+ T cell clusters is not significantly different for the two groups (data not shown). When we base the analysis on raw RECIST criteria regardless of patient cohort and their mismatch-repair deficiency status using Jonckheere's trend test, the trend toward objective response and stable disease is also significant in tumor region in addition to the invasive front (Figure S3). Due to the different implication of stable disease for a mismatch-repair deficient or proficient paitent, using invasive front as opposed to tumor center metrics could lead to improved treatment strategies.

## Discussion

We developed a workflow to evaluate whole slide images of cancer immunohistochemistry and quantitatively expressed their intra- and inter-tumoral spatial heterogeneity using different metrics. We applied the method to image data from MSI and MSS cancer patients, starting with segmentation of CD8+ T cells to map out the coordinates of each cell in the slides. By fitting spatial point process models to a sliding window of the full point pattern, we calculated spatial statistics for different locations in the patient tissue to describe intra-tumoral heterogeneity. We also detected cell aggregates using cluster analysis, and used the calculated shape descriptors for each cluster to capture the intra-tumoral heterogeneity. Inter-tumoral heterogeneity is calculated for each metric from the average value within each patient. Results indiate that compared with the variability of the mean values from individuals among a population, the variability within each patient is even higher. We also tested how the metrics we obtained could be suggestive of anti-tumor immunotherapy treatment outcomes. Our results show that although average CD8+ T cell density in different tissue regions does not correlate with responsiveness, high density clusters, especially the number of circular and elongated clusters, are significantly higher in patients who respond to the treatment. The sample we analyzed is relatively small, but the methods used can be applied to larger patient cohorts where statistical methods could be applied to draw clinically-relevant conclusions by taking into account additional clinical parameters such as patient progression free and overall survival.

In this study, only CD8+ cells are subject to analysis, but the framework can also be applied to multiplex datasets. This will further increase the dimensionality of the analysis, which could lead to novel and more in-depth findings. In order to do this, all three modules, i.e., image segmentation, spatial point pattern analysis, and cell cluster morphometric analysis should be further extended. In this particular dataset, because T lymphocytes have relatively regular shapes compared with cancer cells and CD8+ staining is mostly confined to the membrane, we can effectively segmented these cells using traditional image processing techniques. However, when additional proteins are stained for, it is possible that the boundaries of individual cells may be difficult to determine, due to the irregular expression patterns and levels of certain proteins. In those scenarios, the image segmentation stage could involve utilization of more sophisticated deep learning algorithms trained with the assistance from pathologists for better detection of objects of interest. In the step where we performed spatial point process model fitting, we used a univariate model to analyze the clustering properties of CD8+ T cells. Point pattern analysis of multiple cell types, such as PD-L1+ cells, and Tregs, will open up opportunities to use multivariate point process models to assess interactions between different cell types and quantify these interactions with fitted parameter values (43). The aforementioned extensions of our presented workflow can lead to a deeper understanding of tumor spatial heterogeneity.

By quantifying the inter-tumoral heterogeneity, we are also aiming to evaluate the population scale variability using different metrics. A limitation of this study is the small number of patients involved. The heterogeneity in mismatch repair status among the patients further limit the number of availabe patients within each cohort. In fact, such limitation is almost ubiquitous, considering the vast number of ongoing clinical trials in the field of immuno-oncology. Computational systems biology models, especially spatial agent-based models, is one possible tool to help with this situation. The numerical representation of 2D image data can help with calibration and validation of such spatially explicit systems biology models by ensuring that the heterogeneity is accounted for. Due to the mechanstic nature of such models, after being trained on the relatively small number patients, they can serve as a platform for virtual clinical trials by generating cohorts of simulated patients with *in silico* cancer devleopment dynamics and immunotherapy treatment responses. Using such models, it is possible to investigate the causaility between functionality (which in this study is responsiveness to PD-1 blockade treatment) and mechanisms, gaining insight into the underlysing biological system. By simulating a large number of tumors, such model can also generate spatial patterns in very large numbers (compared to the small patient number used for model parameterization), which can then be fed to a datamining pipeline to help identify predictive biomarkers.

## Author Contributions

CG designed and performed the analysis, interpreted the data, made the figures and drafted the manuscript. RA annotated the pathological data. RA, QZ, JT, and BG contributed to the analysis and interpretation of pathological data. WC helped with statistical analysis. IB, BW, PV, and AP helped design the analysis, interpreted the data, and edited the manuscript.

### Conflict of Interest Statement

IB, PV, and BW were employed by MedImmune, LLC. The remaining authors declare that the research was conducted in the absence of any commercial or financial relationships that could be construed as a potential conflict of interest.
