# Supplementary material for: Quantitative Characterization of CD8+ T Cell Clustering and Spatial Heterogeneity in Solid Tumors
Source: Front Oncol. 2019 Jan 7;8:649. doi: 10.3389/fonc.2018.00649 (PMC6330341; doi:10.3389/fonc.2018.00649)
Supplement: Supplementary file 1 [file Data_Sheet_1.docx]

**Quantitative characterization of CD8+ T cell clustering and spatial heterogeneity in solid tumors**

**Supplement**

**The effect of window size on heterogeneity metrics**

To determine what window size to use in order to best assess the intra-tumoral heterogeneity using spatial statistics method, we use the point pattern from one patient as an example and analyze how window sizes ranging from 0.1 to 10 mm affect the heterogeneity of cluster size obtained from spatial point process model fitting. As shown in Figure S1, the number of windows with aggregated patterns increases initially as the window size increases. With small window sizes, only a very confined sub-region with limited number of cells is included in the analysis, resulting in frequent underestimation of local density variability and failure to pass the complete spatial randomness test. The number of sub-regions with aggregated patterns reaches maximum when window size is around 0.4-0.5 mm, and then decreases because fewer windows are available for analysis as window size increases. For each chosen window size, we calculated the quartile coefficient of dispersion (QCoD) of fitted CD8+ cluster size. The QCoD decreases as window size increases, except when the window size exceeds 2 mm, where the number of available windows becomes small and the statistics become less robust. This is expected, as during the model fitting, the point pattern process within each window is assumed to be stationary. The variability between different windows becomes smaller when each estimation is obtained by averaging over a large window.

The ideal window size should be large enough so that local density variation is properly taken into account, and at the same time small enough so that the spatially stationary assumption does not lead to underestimation of global variability among different windows. Based on these considerations, we decide to use 0.5 mm as the window size for spatial point patter analysis.


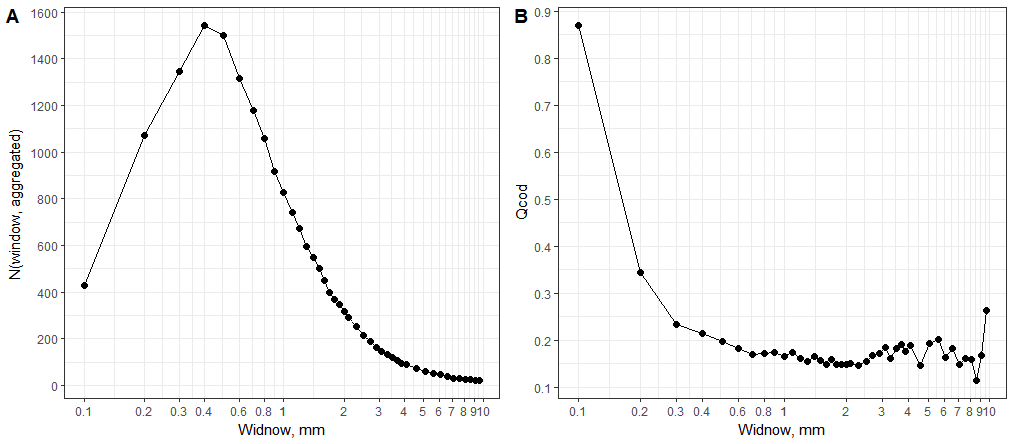


Figure S1. Window size affects intra-tumoral heterogeneity measures in spatial point pattern analysis. A. Tthe number of windows in one slide with aggregated CD8+ T cell patterns using different window sizes. B. Qquartile coefficient of dispersion (QCoD) of fitted cluster radii corresponding to different choices of window sizes.


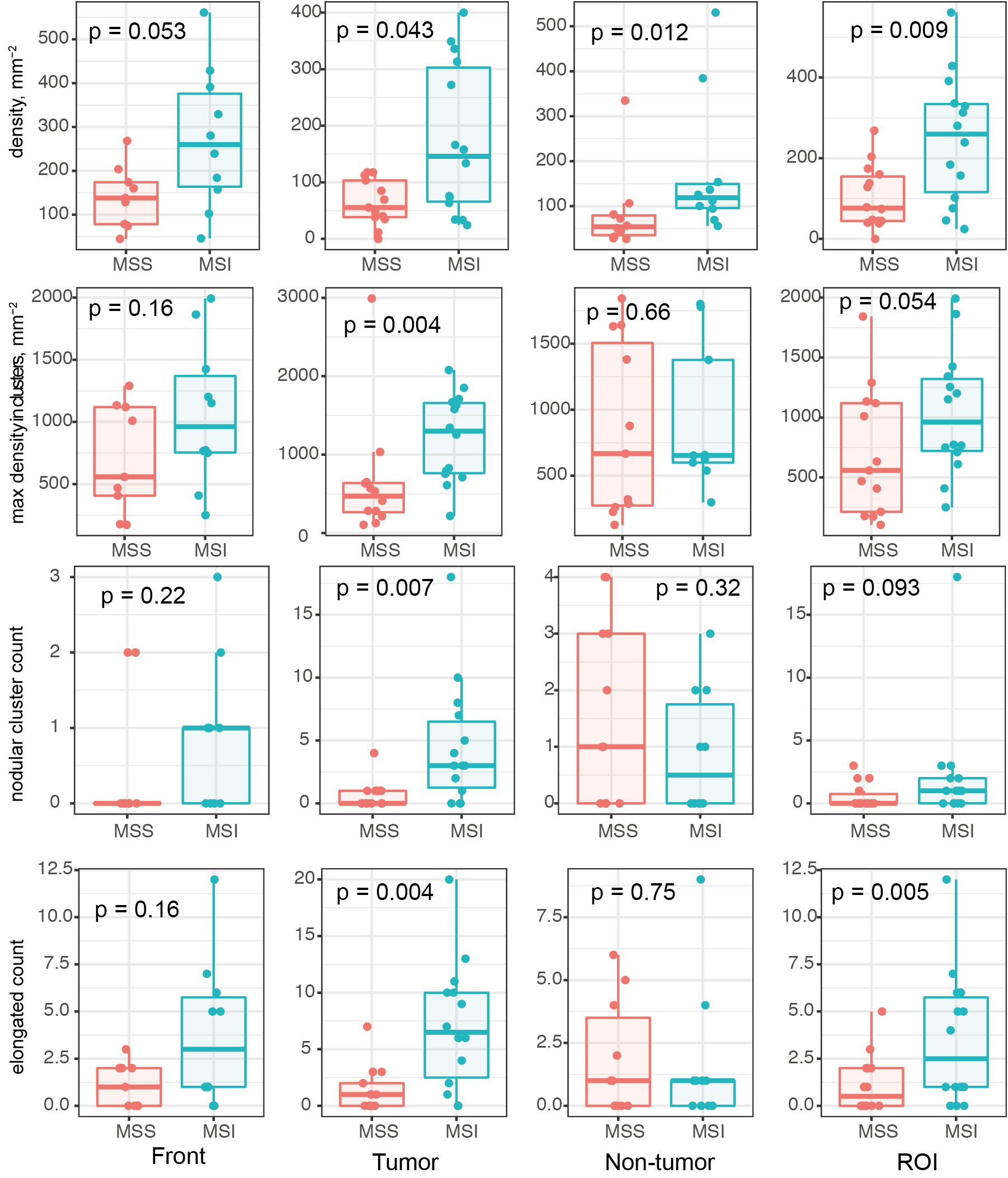


Figure S2. Region specific CD8+ metrics in microsatellite stable and microsatellite instable patients. P-values are obtained using Wilcoxon rank-sum test.


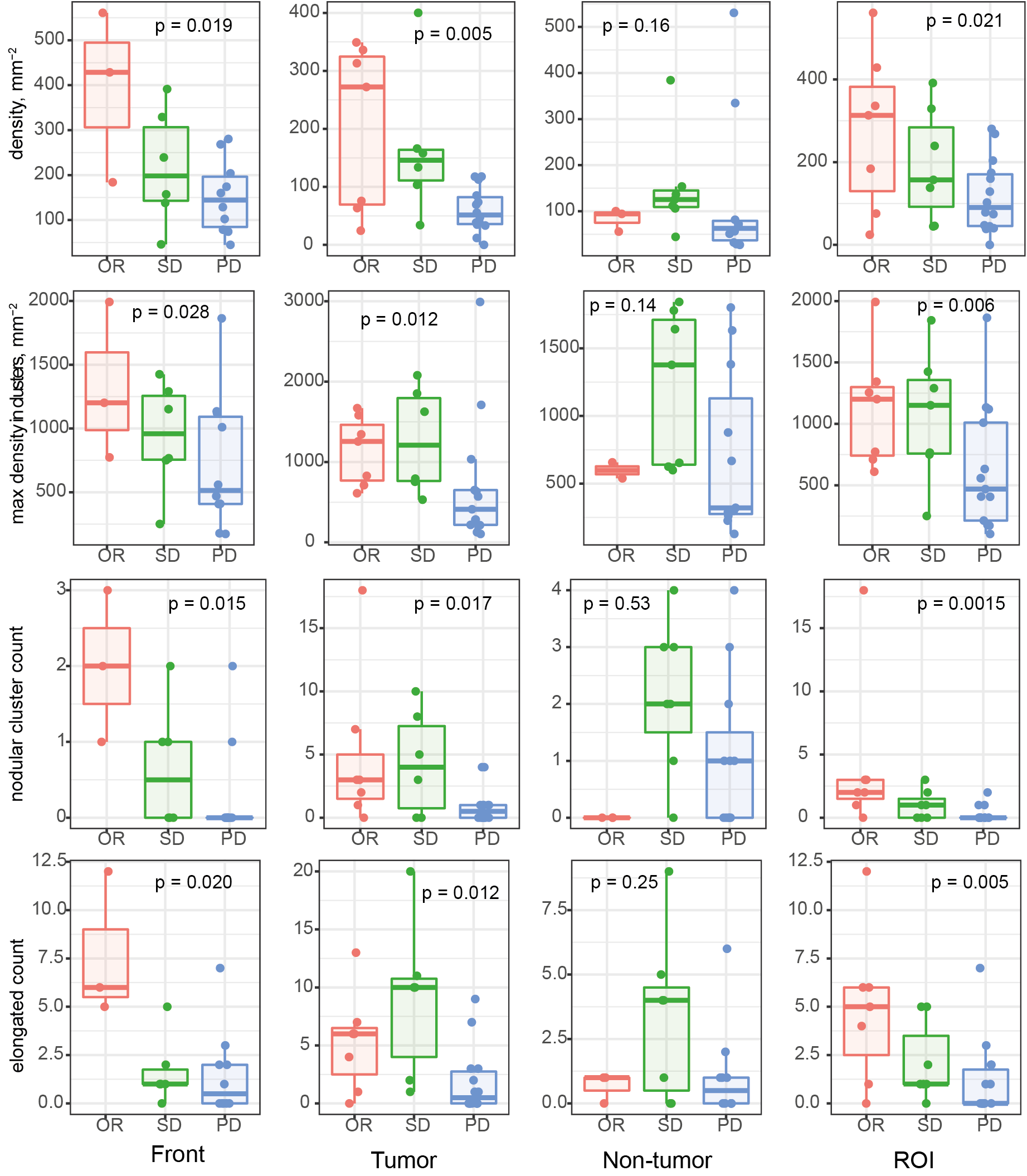


Figure S3. Correlations between region specific CD8+ metrics and objective response to immune checkpoint blockade therapy. Jonckheere trend test is used to evaluate the trend.

.

| Patient ID | Reference ID | Total CD8+ number | CD8+ density  (mm^-2^) | Tissue area (mm^2^) | Front area  (mm^2^) | Tumor area  (mm^2^) | Stroma area  (mm^2^) | ROI | Outcome |
| --- | --- | --- | --- | --- | --- | --- | --- | --- | --- |
| 1 | 29 | 22611 | 310.08 | 72.92 | 0.00 | 67.29 | 0.00 | Tumor | CR |
| 2 | 27 | 21156 | 212.11 | 99.74 | 7.26 | 33.54 | 50.69 | Front | Clinical PD |
| 3 | 31 | 5906 | 60.16 | 98.17 | 0.00 | 78.03 | 0.00 | Tumor | PR |
| 4 | 33 | 29120 | 93.50 | 311.45 | 6.99 | 286.52 | 14.76 | Front | PD |
| 5 | 8 | 9294 | 23.96 | 387.83 | 0.00 | 380.36 | 0.00 | Tumor | PR |
| 6 | 4 | 5938 | 35.59 | 166.84 | 9.80 | 74.99 | 47.80 | Front | PD |
| 7 | 14 | 1429 | 17.78 | 80.38 | 0.00 | 30.21 | 0.00 | Tumor | PD |
| 8 | 26 | 98 | 2.03 | 48.22 | 0.00 | 2.55 | 0.00 | Tumor | Clinical PD |
| 10 | 9 | 34336 | 141.09 | 243.36 | 3.67 | 185.75 | 39.78 | Front | SD |
| 11 | 10 | 25591 | 94.77 | 270.03 | 9.77 | 65.12 | 174.59 | Front | SD |
| 12 | 1 | 61947 | 155.57 | 398.20 | 12.72 | 326.95 | 38.86 | Front | SD |
| 13 | 3 | 4772 | 19.09 | 249.92 | 11.32 | 116.85 | 113.58 | Front | PD |
| 15 | 13 | 1755 | 12.12 | 144.76 | 0.00 | 0.00 | 136.73 | Stroma | PD |
| 16 | 11 | 11294 | 37.49 | 301.29 | 4.43 | 276.98 | 13.48 | Front | SD |
| 17 | 32 | 718 | 2.74 | 262.09 | 0.00 | 17.87 | 0.00 | Tumor | PD |
| 18 | 41 | 11371 | 41.02 | 277.21 | 12.18 | 140.90 | 121.55 | Front | Clinical PD |
| 19 | 30 | 42086 | 208.23 | 202.11 | 12.99 | 124.02 | 69.43 | Front | PR |
| 20 | 18 | 17514 | 76.74 | 228.23 | 9.91 | 135.98 | 67.00 | Front | PD |
| 21 | 16 | 106271 | 356.96 | 297.71 | 9.95 | 170.10 | 97.31 | Front | SD |
| 22 | 15 | 18035 | 39.56 | 455.93 | 18.81 | 237.82 | 34.01 | Front | PR |
| 23 | 17 | 4872 | 26.45 | 184.22 | 8.05 | 105.62 | 19.41 | Front | PD |
| 24 | 28 | 27193 | 78.99 | 344.24 | 11.17 | 138.01 | 186.11 | Front | PD |
| 25 | 25 | 3040 | 295.74 | 10.28 | 0.00 | 9.71 | 0.00 | Tumor | PR |
| 26 | 19 | 55274 | 207.07 | 266.93 | 17.01 | 120.33 | 118.73 | Front | PR |
| 27 | 21 | 18007 | 67.99 | 264.86 | 13.54 | 106.52 | 136.04 | Front | PD |
| 28 | 38 | 16248 | 51.93 | 312.88 | 11.97 | 66.62 | 229.53 | Front | Clinical PD |
| 29 | 22 | 10591 | 42.99 | 246.35 | 0.00 | 0.00 | 238.00 | Stroma | SD |
| 31 | 36 | 42603 | 115.71 | 368.19 | 8.28 | 250.43 | 76.07 | Front | SD |

Table S1. Patient CD8+ T cell segmentation, tissue annotation, and treatment outcomes. Reference IDs are subject IDs used in Le et al., 2015
